# Milk: An Effective Recovery Drink for Female Athletes

**DOI:** 10.3390/nu10020228

**Published:** 2018-02-17

**Authors:** Paula Rankin, Adrian Landy, Emma Stevenson, Emma Cockburn

**Affiliations:** 1 Department of Science and Health, Institute of Technology, Carlow R93 V960, Ireland; adrianlandy@gmail.com; 2 London Institute of Sport, Middlesex University, London NW4 4BT, UK; emma.cockburn@ncl.ac.uk; 3 School of Biomedical Science, Newcastle University, Newcastle Upon Tyne NE2 4HH, UK; 4 Institute of Cellular Medicine, Newcastle University, Newcastle Upon Tyne NE2 4HH, UK; emma.stevenson@ncl.ac.uk

**Keywords:** milk, protein, muscle damage, recovery, female

## Abstract

Milk has become a popular post-exercise recovery drink. Yet the evidence for its use in this regard comes from a limited number of investigations utilising very specific exercise protocols, and mostly with male participants. Therefore, the aim of this study was to investigate the effects of post-exercise milk consumption on recovery from a sprinting and jumping protocol in female team-sport athletes. Eighteen females participated in an independent-groups design. Upon completion of the protocol participants consumed 500 mL of milk (MILK) or 500 mL of an energy-matched carbohydrate (CHO) drink. Muscle function (peak torque, rate of force development (RFD), countermovement jump (CMJ), reactive strength index (RSI), sprint performance), muscle soreness and tiredness, symptoms of stress, serum creatine kinase (CK) and high-sensitivity C-reactive protein (hsCRP) were determined pre- and 24 h, 48 h and 72 h post-exercise. MILK had a *very likely beneficial* effect in attenuating losses in peak torque (180°/s) from baseline to 72 h (0.0 ± 10.0% vs. −8.7 ± 3.7%, MILK v CHO), and countermovement jump (−1.1 ± 5.2% vs. −10.4 ± 6.7%) and symptoms of stress (−13.5 ± 7.4% vs. −18.7 ± 11.0%) from baseline to 24 h. MILK had a *likely beneficial effect* and a *possibly beneficial* effect on other peak torque measures and 5 m sprint performance at other timepoints but had an *unclear* effect on 10 and 20 m sprint performance, RSI, muscle soreness and tiredness, CK and hsCRP. In conclusion, consumption of 500 mL milk attenuated losses in muscle function following repeated sprinting and jumping and thus may be a valuable recovery intervention for female team-sport athletes following this type of exercise.

## 1. Introduction

Bovine milk, which comprises both whey and casein protein along with carbohydrate, lipids, vitamins and minerals, has become a popular post-exercise recovery drink. It’s carbohydrate content (as lactose) is comparable to commercial sports drinks and may be beneficial in post-exercise glycogen synthesis [1]. It’s whey and casein protein content enhances post-exercise muscle protein synthesis rates [2] while the high concentration of electrolytes facilitates fluid recovery following exercise [3]. 

Recent research has shown that the consumption of 500 mL milk following muscle damaging exercise can attenuate decreases in muscle function in males [4,5] and females [6] including peak torque, reactive strength index (RSI) and sprint performance. These studies evoked muscle damage using an intense mechanical eccentric protocol on an isolated muscle group. Recently, Rankin et al. [7] reported that milk was of little benefit in recovery from repeat-sprint cycling (a predominantly concentric exercise) in female athletes, suggesting that the benefit of milk for athletic recovery is likely to be greatest following activities that have an eccentric component. Given that the mechanisms underpinning recovery following eccentrically biased and concentrically biased exercise may be different, it follows that choosing nutritional interventions that are specific to the mode of exercise and desired outcomes is required, and as recently discussed a ‘one size fits all’ approach is inappropriate [8]. Conceptualising a continuum of exercise from isolated mechanical eccentric exercise to isolated metabolic concentric exercise, the vast amount of exercise activities reside somewhere between, inducing both mechanical and metabolic stress to varying degrees. Participation in many sports and exercise activties involves repeated high-intensity activity such as sprinting and jumping, change of direction, and deceleration [9,10]. Thus, for the purpose of exploring post-exercise recovery, opportunities exist for research utilising more sport-specific models which have greater ecological validity and practical significance. This has been addressed in investigations employing sprinting, [11], jumping [12] and drop-jumping damage protocols [13], which reported reductions in isometric strength, CMJ and squat jump performance, along with increased CK and muscle soreness. No investigations have been conducted utilising combined sprinting and jumping activities. Moving forward, the key strategy must be to match recovery strategies with the specific demands of exercise and thus research exercise protocols must incorporate relevant activities.

Of significance, the aforementioned studies [11,12,13] were conducted with male participants. Our previous research has suggested gender differences in the response to eccentric exercise with females showing smaller increases in sprint time, passive soreness, active soreness and sTnI values [6]. Thus, this reinforces the necessity of matching recovery interventions not only to the nature of the exercise demands but also to the population group. There is undoubtedly a dearth of research focusing on reponses of female athletes to varied forms of exercise. Indeed, a recent study concluded that female participants are under-represented in sports and exercise research, comprising only 39% of participants across 1382 investigations [14]. In an informative study Keane et al. [15] investigated muscle damage in female athletes following a repeat-sprint protocol incorporating 15 × 30 m sprints and reported reductions in maximal voluntary isometric contraction (MVIC), countermovement jump height and sprint performance alongside increases in muscle soreness and serum creatine kinase over the 72 h recovery period. Comparable results have been reported with female dancers following the same protocol [16]. Similarly Jakeman et al. [17] noted reduced squat jump height, CMJ height, isokinetic muscle strength alongside increased muscle soreness and CK in physically active females following the execution of 10 × 10 repetitions of drop jumps. While these investigations contribute to our understanding of responses to eccentrically loaded exercise in female athletes, there are few investigations examining nutritional interventions following exercise in females, and none exploring the effect of milk on recovery following this type of exercise. 

Accordingly, specific strategies to ameliorate the deleterious effects of exercise and muscle damage and accelerate the recovery process are of importance to researchers, coaches and athletes. No studies have examined the potential of milk to enhance recovery from combined sprinting and jumping activities, yet this knowledge would be valuable for post-exercise nutritional prescription for athletes. Moreover, establishing responses in female athletes can present new information on recovery strategies for this under-investigated population. Therefore, the aim of this study was to examine the effect of milk on recovery from repeated sprinting and jumping in a female team-sport population. 

## 2. Materials and Methods 

### 2.1. Participants

Eighteen female team-sport (camogie and ladies gaelic football) athletes (mean age 21.6 ± 3.4 y), training a minimum of twice per week, participated in this study. Mean (±SD) height and mass were 162.9 ± 9.3 cm and 64.3 ± 6.1 kg, respectively. Participants completed a health screening questionnaire and were excluded if they met any of the following: intolerance to dairy or lactose products, lower limb or back injury in the previous 3 months, surgery in the previous 6 months, known coronary disease, uncontrolled metabolic or respiratory disease, pregnancy, post-partum or breastfeeding. Participants were provided with verbal and written briefings, following which written informed consent was recorded. Ethics approval was provided by the London Sport Institute Ethics Sub-Committee at Middlesex University (Application 0432), and the Institute of Technology Carlow (Reference Number: 134), where the data collection took place. 

### 2.2. Study Design

The study utilised an independent group design that required participants to visit the laboratory on six occasions. The initial visit comprised familiarisation and the subsequent visit, no more than 3 days later, involved the determination of baseline (B) values for dependent variables. Participants were then randomly but equally divided into two groups: Milk (MILK) and carbohydrate (CHO). Completion of the exercise protocol took place no more than 5 days after familiarisation, and participants returned to the laboratory 2 h (bloods only) 24 h, 48 h and 72 h post-exercise for re-evaluation of the dependent variables. Visits took place at the same time of day preceded by an overnight fast and abstinence from alcohol, caffeine and exercise. Participants were requested to refrain from any strenuous activity for the duration of the study, and from treating symptoms of muscle soreness and tiredness with interventions such as massage, cryotherapy, supplements and non-steroidal anti-inflammatory drugs.

### 2.3. Nutritional Intervention and Dietary Control

Immediately upon completion of the protocol participants consumed either 500 mL of milk (Avonmore 1% Light, Glanbia, Kilkenny, Ireland) or 500 mL of an energy-matched carbohydrate solution. The consumption of carbohydrate drinks post-exercise is common practice and previous research has demonstrated that the administration of carbohydrate alone has no effect in lessening signs of EIMD [18]. Macronutrient composition (per 500 ml) of milk was: Energy 910 kJ/215 kcal, Protein 17.0 g, Carbohydrate 25.5 g, Fat 5.0 g. A volume of 500 mL was chosen based on previous research [4,5]. From an applied perspective, it was felt that 500 mL was an easily consumed volume and that consumption of larger volumes may lead to stomach fullness and discomfort. The energy-matched carbohydrate solution (Energy 910 kJ/215 kcal, Protein 0.2 g, Carbohydrate 53.6 g, Fat 0.0 g) consisted of glucose mixed with water and a commercially available orange flavoured cordial (Nutritional information per 100 mL of cordial: Energy 37 kJ/9 kcal, Protein 0.2 g, Carbohydrate 0.8 g, Sodium Trace; MiWadi, Dublin, Ireland). Participants completed a food diary for 24 h prior to testing and for the subsequent 3 days which was analysed using dietary analysis software (Nutritics Professional Diet Analysis, Nutritics Ltd., Dublin, Ireland). Analysis using Magnitude Based Inference indicated that there were no clear differences in energy, carbohydrate, protein or fat intake between groups.

### 2.4. Exercise Protocol

The aim of the exercise protocol was to incorporate whole-body sport and exercise activities. Participants completed a 10 min warm-up consisting of self-paced jogging, sprint drills and sprints at 60%, 80% and 100% of perceived maximum speed, followed by a series of dynamic stretches [11]. Participants then lined up at a marker 30 cm from the start line and completed 15 × 20 m maximal sprints stopping within a 10 m deceleration zone marked out at the end of each sprint. Previous research has found that a similar protocol (15 × 30 m) has resulted in EIMD in females [15]. Following a 5 min rest period, participants completed eight sets of 10 plyometric jumps, a protocol which has previously shown to induce muscle damage in males [12]. With feet shoulder width apart and hands on hips, participants were asked to flex their knees to 90 degrees, and to jump as high as possible on each jump. Standardised verbal encouragement was provided to participants throughout the protocols. 

### 2.5. Blood Sampling

On each day, prior to other measures and following 10 min rest in the supine position, a venous blood sample was obtained via venepuncture from a forearm vein at the antecubital fossa and anlayzed for CK and hsCRP using high sensitivity procedures (Roche Cobas 6000 chemistry module c501, Hoffmann-La Roche, Basel, Switzerland). Coefficients of variation for this system are reported as 0.7–2.3%.

### 2.6. Peak Torque

Peak torque was determined as previously described [6]. Following a standardised warm-up, participants completed three maximal effort knee extension and flexion repetitions at 60°/s and 180°/s on the dominant leg, with 60 s seated recovery between speeds on a Biodex System3 Isokinetic dynamometer (Biodex Medical System, New York, NY, USA). Participants were instructed to give maximal effort and to complete full range of motion for each repetition. Interclass correlations for this protocol at IT Carlow are 0.83–0.94.

### 2.7. Rate of Force Development

Rate of force development (RFD) over the first 200 ms of an isometric contraction was determined as previously described [19]. Two maximal 5 s isometric contractions of the dominant leg quadriceps were performed with the knee fixed at an angle of 70°. Participants were instructed to contract and ‘push away as fast and forcefully as possible’. RFD was calculated over the time interval of 0–200 ms (Δtorque/Δtime) relative to the onset of contraction, which was defined as the time point when the torque generated exceeded the baseline by >7.5 Nm [19]. 

### 2.8. Countermovement Jump Height

Countermovement jump height (cm) was determined using an Optojump optical measurement system (Microgate, Bolzano, Italy). Participants completed three trials employing standard countermovement jump technique, commencing the movement in the upright position with hands on the hips, then flexing the knees to approximately 90° and immediately jumping for maximal height. The highest recorded jump was used for analysis. Interclass correlations from reliability trials at IT Carlow for this protocol are 0.97–0.98.

### 2.9. Reactive Strength Index

Reactive Strength Index (RSI) was measured using the Optojump optical measurement system (Microgate, Bolzano, Italy). Participants performed three maximal effort drop jumps from a height of 45 cm onto an indoor sprint track. Participants were instructed to minimise ground contact time when landing from the drop height, while maximising subsequent jump height. Hands were maintained on the hips for the duration of the jump to eliminate any contribution of arm swing. RSI was calculated by dividing jump height (cm) by ground contact time (ms) [20]. 

### 2.10. Sprint Performance

Five metre, 10 m and 20 m sprint performance, from a standing start 20 cm behind the start line, were performed on an indoor sprint track and were recorded using timing gates (Microgate Racetime 2, Bolzano, Italy). Participants were instructed to sprint through the timing gates as fast as possible and each participant completed three sprints with a rest time of 120 s between sprints. Interclass correlations from reliability trials at IT Carlow for this protocol are 0.89–0.98.

### 2.11. Muscle Soreness and Muscle Tiredness

Active muscle soreness was measured on a Visual Analogue Scale (VAS) during squatting to approximately 90° knee flexion and during isokinetic testing of knee extension at 60°/s, with participants rating their level of soreness on a scale of 0 (no soreness) to 10 (as bad as could be). A similar VAS was used to measure passive muscle tiredness, with 0 indicating no tiredness, and 10 indicating as tired as could be. 

### 2.12. Symptoms of Stress

Symptoms of stress were monitored at the same time each day using the validated Daily Analysis of Life Demands (DALDA) questionnaire [21]. The questionnaire is comprised of two parts; Part A represents the sources of stress, and Part B assesses the manifestation of this stress in the form of symptoms. Participants marked each question as being either ‘normal’, ‘worse than normal’, or ‘better than normal’. Data for Part B is presented in this study.

### 2.13. Data Analysis

Data was analysed by making probabilistic magnitude based inferences about the true values of the effect of intervention on outcomes by expressing the uncertainty as 90% confidence limits (CL) as described by Batterham and Hopkins [22]. Within-group effects over time and the effect of MILK versus CHO were determined using published spreadsheets [23]. The smallest standardised (Cohen) difference in the mean (0.2 times the between-subjects standard deviation for all participants) was used to identify the magnitude of the smallest substantive effect. Comparisons were made between baseline and 2 h, 24 h, 48 h, and 72 h post-exercise. Data for peak torque, RFD, countermovement jump, RSI and sprint performance were log-transformed to overcome heteroscedastic error [24]. Soreness values were not log-transformed because of interval scaling [24]. Means of log-transformed data were then back transformed to provide mean percentage change and percentage SD. Serum markers are reported as factors because of large percentage changes [25]. Chances of change over time and of benefit and harm were assessed qualitatively as follows: <1% almost certainly none, 1–5% very unlikely, 5–25% unlikely, 25–75% possibly, 75–95% likely, 95–99% very likely, >99% almost certainly [26]. An effect was deemed unclear if the confidence interval overlapped the thresholds for positive and negative effects. Effect Size (ES) magnitudes were calculated as the difference in means/SD for both groups and were qualified as follows: trivial, 0.0–0.2; small, 0.2–0.6; moderate, 0.6–1.2; large, 1.2–2.0; very large, 2.0–4.0; extremely large, 4.0. [26]. ES thresholds for measures of soreness and tiredness were set at 10%, 30%, 50%, 70% and 90% of the VAS range for small, moderate, large, very large and extremely large, respectively. 

## 3. Results

### 3.1. Within-Group Effects

The exercise protocol employed resulted in Exercise Induced Muscle Damage (EIMD) for all participants. Analysis of within-group effects revealed post-exercise decreases in peak torque, CMJ, sprint performance and RFD, increases in serum CK and hsCRP and in muscle soreness and tiredness. Mean effects, ±90% CI, with qualitative inferences, are presented in Table S1 (Supplementary Material).

### 3.2. Peak Torque

Baseline peak torque values for knee extension at 60°/s for MILK and CHO were 164.4 ± 17.9 Nm and 152.8 ± 10.0 Nm respectively. Changes in peak torque between baseline and 24 h for MILK and CHO were −8.1 ± 3.9% and −12.9 ± 9.3% respectively, a likely small benefit for MILK (ES = 0.38). Unclear outcomes for MILK versus CHO were found at B-48 h (−8.4 ± 4.2% vs. −11.9 ± 11.0%) and B-72 h (−3.9 ± 6.9% vs. −9.8 ± 11.6%). 

At baseline the peak torque values at 60°/s for knee flexion for MILK and CHO were 83.4 ± 16.5 Nm and 72.3 ± 11.2 Nm respectively. Changes in peak torque between baseline and 24 h for MILK and CHO were −4.4 ± 13.3%, and −13.6 ± 10.6% respectively, a possible small benefit of milk (ES = 0.22). There was an unclear outcome for MILK (−9.4 ± 14.2%) versus CHO (−12.9 ± 8.8%) at B-48 h. A likely small benefit (ES = 0.31) was observed for MILK (−2.7 ± 11.7%) versus CHO (−11.8 ± 11.6%) at B-72 h.

**Figure 1:**
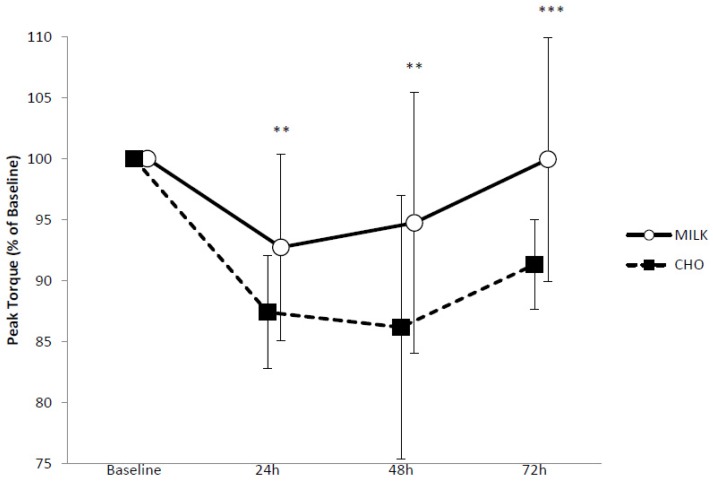
Peak torque at 180°/s for dominant knee extension in response to repeated sprinting and jumping for MILK (*n* = 9) and CHO (*n* = 9). Values are presented as means ± SD. ** Likely benefit of MILK. *** Very likely benefit of MILK.

Pre-exercise mean peak torque values for knee extension at 180°/s were 118.6 ± 14.5 Nm and 109.8 ± 7.8 Nm for the MILK and CHO conditions respectively. A likely small benefit (ES = 0.50) for MILK versus CHO was found at B-24 h (−7.3 ± 7.6% vs. −12.6 ± 4.6%), a likely moderate benefit B-48 h (ES = 0.84) (−5.3 ± 10.7% vs. −13.8 ± 10.8%) and a very likely moderate benefit (ES = 0.94) for MILK from B-72 h (0.0 ± 10.0% vs. −8.7 ± 3.7%). Changes in peak torque can be seen in Figure 1.

Baseline peak torque values for knee flexion at 180°/s for MILK and CHO were 64.4 ± 15.5 Nm and 62.2 ± 11.3 Nm. Changes in the peak torque between baseline and 24 h for MILK and CHO were −2.2 ± 12.4%, and −13.2 ± 11.2% respectively, a likely small benefit (ES = 0.51) of milk. There was an unclear outcome for MILK (−3.4 ± 13.8%) versus CHO (−10.0 ± 16.3%) at B-48 h. A likely small beneficial effect (ES = 0.41) was observed for MILK (−0.8 ± 14.2%) compared to CHO (−10.3 ± 9.4%) at B-72 h.

### 3.3. Rate of Force Development

Baseline values for mean rate of force development were 555.1 ± 294.2 Nm·s^−1^ and 634.3 ± 255.1 Nm·s^−1^ for MILK and CHO respectively. A likely trivial benefit (ES = 0.03) for MILK versus CHO was found at B-24 h (−8.5 ± 16.8% vs. −19.4 ± 15.0%) though unclear outcomes were observed at B-48 h (−16.6 ± 14.3% vs. −20.2 ± 19.1%) and B-72 h (−18.8 ± 36.6% vs.−13.8 ± 21.3%).

### 3.4. Countermovement Jump Performance

**Figure 2:**
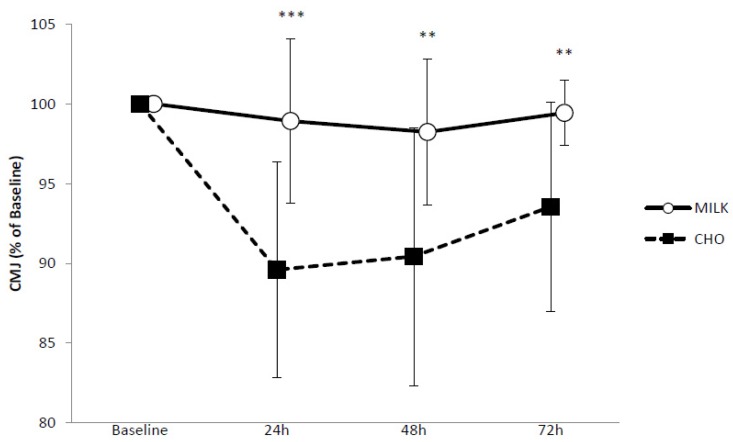
Countermovement jump performance in response to repeated sprinting and jumping for MILK (*n* = 9) and CHO (*n* = 9). Values are presented as means ± SD. *** Very likely benefit of MILK. ** Likely benefit of MILK.

Baseline mean countermovement jump heights for MILK and CHO were 26.7 ± 2.7 cm and 26.7 ± 3.5 cm respectively. A very likely moderate benefit (ES = 0.70) for MILK was seen at B-24 h (−1.1 ± 5.2% vs. −9.7 ± 15.1%), a likely moderate benefit (ES = 0.67) from B-48 h (−1.8 ± 4.6% vs. −9.6 ± 8.1%) and a likely small benefit from B-72 h (−0.6 ± 2.1% vs. −10.0 ± 13.7%, ES = 0.47). Changes in CMJ performance can be seen in Figure 2.

### 3.5. Reactive Strength Inde

Baseline RSI for the MILK group was 105.1 ± 21.9 cm·s^−1^ and for the CHO group was 104.0 ± 24.0 cm·s^−1^. Comparisons of MILK and CHO at B-24 h (−6.7 ± 7.7% vs. −9.7 ± 15.1%), B-48 h (−12.3 ± 10.6% vs. −12.6 ± 18.7%) and B-72 h (−4.7 ± 12.5% vs. −10.0 ± 13.7%) revealed unclear outcomes. 

### 3.6. Sprint Performance

At baseline, 5 m sprint times were 1.23 ± 0.10 s and 1.25 ± 0.07 s for MILK and CHO, respectively. Between baseline and 24 h there was a possible small benefit (ES = 0.45) for MILK (−1.6 ± 3.4%) compared to CHO (−4.5 ± 4.5%). Comparisons of MILK and CHO B-48 h (0.5 ± 6.3% vs. −2.9 ± 4.4%) and B-72 h (−0.5 ± 4.9% vs. −1.3 ± 3.2%) were unclear. Baseline 10 m sprint times for MILK and CHO were 2.06 ± 0.12 s and 2.09 ± 0.08 s. All MILK vs. CHO comparisons gave an unclear outcome (B-24 h −1.1 ± 3.0% vs. −2.3 ± 4.6%; B-48 h 0.0 ± 4.0 vs. 1.8 ± 4.4%; B-72 h −0.2 ± 4.8% vs. −0.4 ± 4.5%). Similarly, with baseline values of 3.57 ± 0.18 s (MILK) and 3.61 ± 0.11 s (CHO), unclear outcomes were observed for 20 m sprint performance (B-24 h −1.2 ± 2.0% vs. −2.6 ± 3.6%; B-48 h −0.6 ± 1.9% vs. −2.0 ± 4.4%; B-72 h −0.6 ± 2.1% vs. −0.1 ± 2.8%). 

A summary of the statistical analysis for Peak torque, RFD, CMJ, RSI and sprint performance can be seen in Table 1. 

### 3.7. Muscle Soreness and Tiredness

Peak soreness and tiredness was observed 24 h following exercise, and values had not returned to baseline by 72 h. A likely trivial benefit for MILK (27.1 ± 13.8%) versus CHO (31.7 ± 16.6%) was observed at 72 h for soreness during isokinetic testing; all other comparisons were unclear. 

### 3.8. DALDA

For DALDA B a very likely small benefit of MILK (13.5 ± 7.4%) compared to CHO (18.7 ± 11.0%) was observed at B-24 h and a likely small benefit was observed at B-48 h (15.5 ± 10.1% vs. 19.6 ± 14.1%). From B-72 h (11.0 ± 11.5% vs. 12.9 ± 10.5%) outcomes were unclear.

### 3.9. Creatine Kinase

Baseline serum CK values were 128.5 ± 118.4 U/L and 192.2 ± 103.6 U/L for MILK and CHO respectively. Unclear outcomes for the comparison of MILK versus CHO were found at B-2 h (1.8 ×/÷ 1.3 vs. 1.7 ×/÷ 1.3), B-24 h (2.3 ×/÷ 1.8 vs. 2.1 ×/÷ 1.4), B-48 h (1.8 ×/÷ 1.9 vs. 1.4 ×/÷ 1.8) and B-72 h (1.4 ×/÷ 1.9 and 1.1 ×/÷ 1.8).

### 3.10. hsCRP

Baseline hsCRP values were 1.0 ± 0.6 mg/L and 1.3 ± 0.6 mg/L for MILK and CHO. Unclear outcomes were observed for MILK versus CHO at B-2 h (1.1 ×/÷ 1.2 vs. 1.0 ×/÷ 1.3), B-24 h (1.3 ×/÷ 2.0 vs. 1.0 ×/÷ 1.3), B-48 h (1.2 ×/÷ 2.0 vs. 0.9 ×/÷ 1.3) and B-72 h (1.1 ×/÷ 1.9 vs. 0.8 ×/÷ 1.5). Mean effects for all measures of soreness and tiredness, symptoms of stress, CK and hsCRP can be seen in Table 2.

## 4. Discussion

The key findings of this investigation indicate that consumption of 500 mL of milk post-repeated sprinting and jumping gives small to moderate benefits in attenuating losses in CMJ and peak torque at 60° and 180°/s for both extension and flexion, similar to previous research with female athletes following maximal eccentric contractions [6]. Additionally, a benefit for MILK was seen for RFD (trivial) and 5 m sprint (small) over the first 24 h of recovery, but outcomes for RSI, 10 m or 20 m sprint performance, muscle soreness and tiredness, CK and hsCRP were unclear. 

There are a number of possible explanations for the observed benefits of milk on muscle function. The composition of milk, with ~80% casein and ~20% whey protein, provides both fast and slow delivery of amino acids for muscle protein turnover [2]. The consumption of protein early in the recovery period is likely to increase the phosphorylation of signalling proteins that regulate translation initiation and elongation, thus enhancing muscle protein synthesis [27]. Furthermore, it is possible that milk has a positive effect on the calpain degredative pathways that are initiated following eccentric exercise consequential to increased cellular concentrations of calcium [28]. This would result in a preservation of myofibrillar and membrane protein integrity and force transmission ability, and an attenuation of losses in muscle function during the recovery period. Moreover, increased insulin levels have been observed following the consumption of milk [29]. Raised insulin levels inhibit muscle protein breakdown [30] perhaps via the ubiquitin-proteasome pathway, further contributing to preservation of muscle function following EIMD.

The reasons for disparity in results between peak torque, RFD, 5 m sprint and measures of RSI, 10 m and 20 m sprint are uncertain. The preferential damage of type 2 fibres with eccentric loading leads to reduced ability to generate power [31] which is key in the explosive nature of the assessment of RFD and 5 m sprint performance; the consumption of milk was beneficial in attenuating these losses. RSI is an assessment of the stretch shortening cycle (SSC). Twist and Eston [32] proposed that a reduced reflex sensitivity during the SSC following EIMD could impair the ability to use ground impact forces, such as during drop jumping. It is conceivable that milk does not have an effect on this reduced neuromuscular function following EIMD, though this does not explain a lack of benefit of milk on 10 m and 20 m sprint performance. Given that the outcomes were unclear, further investigation, with larger sample sizes, may provide greater insight.

There was an unclear effect of milk on serum CK concentration. Muscle membrane damage following eccentric loading occurs as a result of the mechanical stress during the primary phase of muscle damage with further disruption via the lysosomal pathway during the secondary phase [33]. The lack of a clear intervention effect on serum CK suggests that the consumption of milk may not affect this pathway. CK concentration, a marker of scarcolemma damage, was elevated during the recovery period, peaking 24 h post-exercise, and returning to baseline values by 72 h. Mean peak CK values (246.4 ± 75.8 and 373.9 ± 157.82 U/L for MILK and CHO respectively) were lower than observed for female athletes in an investigation employing isolated eccentric exercise [6], higher compared to those reported following repeat-sprint cycling with females [7] but comparable to values reported following repeated sprinting in females [15]. This is not surprising considering that the first investigation provoked muscle damage on an isolated muscle group utilising a maximal isokinetic eccentric loading while the second utilised a protocol lacking eccentric loading. The reported CK response is lower than that reported following similar exercise in males [11] though not unexpected as it has been suggested that females experience reduced post-exercise muscle damage compared to males because of a protective effect of oestrogen [34]. The observed beneficial effect of milk on muscle function and unlear effect on CK implies that serum CK is not a good indicator of muscle function following exercise, a conclusion alluded to most recently by Scott et al. [35] following participation in a soccer match.

A comparison of conditions demonstrates little benefit for MILK compared to CHO in the perception of muscle soreness, though given that DOMS is a subjective measure, it is difficult to compare independent groups. Increases in soreness were lower than those reported in previous studies following repeated sprinting [11] and repeated jumping [12] However, these studies utilised male participants, and oestrogen may have an effect on the perception of pain and level of muscle soreness experienced following damaging exercise by playing a role in the binding of opioids to receptors in the brain and spinal cord thereby modifying the transmission of pain [36]. Nonetheless, the observed soreness ratings were also lower than those reported by Keane et al. [15] following a repeat-sprint protocol with females, despite the addition of repeated jumping in the current study. The reasons for this are unclear, though may reflect different degrees of prior exposure to repeated sprinting and jumping. Similar to the female participants in this study, those in Keane et al. were regular participants in team sport training and competition, though in soccer, rugby union and netball. The participants in the current study were Gaelic football and camogie players and while the demands of these sports are somewhat similar to other team sports the differences may contribute to the difference in the responses to the exercise protocol. In the current study hsCRP levels did not increase following exercise despite an increase in soreness and inflammation being one proposed explanation for soreness. However, serum hsCRP levels may not be reflective of the total cytokine activity in the post-exercise period. The disparity of muscle function and soreness results observed indicate different pathways for the recovery of muscle function and perception of soreness, which may or may not involve the inflammatory response. Similar to CK, muscle soreness is a poor indicator of muscle function during the recovery period. 

The measurement of symptoms of stress was completed using the DALDA questionnaire. Analysis indicated that those who consumed milk reported fewer ‘worse than normal’ symptoms 24 h post-exercise. Physiological processes can be influenced by psychological status [37], and it is known that an individual’s belief in the efficacy of an intervention can influence subsequent responses [38]. It is possible that this was the case in the current study as the participants were not blinded to the recovery drink received and may have been aware of potential benefits of milk for recovery through previously published research. However, given that there were no differences in perception of soreness or tiredness suggests genuine reduced symptoms of stress in the participants who consumed milk.

A limitation of the study is that there was no control for phase of the menstrual cycle, and thus participants were likely to have had varying levels of circulating hormones, including oestrogen as mentioned above, at the time of testing. Nonetheless, it has been reported that oestrogen concentration may have limited effect on the symptoms of EIMD in exercising females [39]. It is possible that a larger intake of milk may have provided additional recovery benefits. While previous research has reported recovery benefits with consumption of 500 mL milk [4,5,6], this volume provides 17 g of protein which is lower than the recommended 20 g threshold for maximal stimulation of protein synthesis [40], and substantially lower than the 40 g recently found to be of greater benefit than 20 g following whole-body exercise [41]. Furthermore, given that post-exercise protein synthesis remains elevated for at least 24 h post-resistance exercise [42] additional prescribed intake of milk during this time period may influence recovery. Research opportunities exist to extrapolate this further, particularly regarding recovery from team-sport specific exercise and in a female cohort. 

## 5. Conclusions

In conclusion, the consumption of 500 mL of milk post repeated sprinting and jumping had a positive effect on the attenuation of losses in muscle function, thus improving recovery, compared to an energy-matched carbohydrate drink. From a practical perspective, this intervention may serve to enhance the recovery of female team-sport athletes following activities that involve sprinting and jumping. This study reinforces the necessity for specific recovery interventions that are matched to the mode of exercise, in this case when the exercise includes eccentric loading. As is evident from this study, including milk in such a mode-specific approach is beneficial for recovery in female athletes. 

## Figures and Tables

**Table 1:** Effects on muscle function following repeated sprinting and jumping.

Variable	Time Frame	Mean Effect ^a^, ±90% CI ^b^	Qualitative Inference ^c^
Peak Torque 60°/s Dominant leg extension	B-24 h	6.0, ±7.7	Likely beneficial
	B-48 h	4.6, ±8.7	Unclear
	B-72 h	7.2, ±10.3	Unclear
Peak Torque 60°/s Dominant leg flexion	B-24 h	8.7, ±13.5	Possibly beneficial
	B-48 h	3.3, ±13.4	Unclear
	B-72 h	9.4, ±12.3	Likely beneficial
Peak Torque 180°/s Dominant leg extension	B-24 h	7.2, ±6.4	Likely beneficial
	B-48 h	10.8, ±9.6	Likely beneficial
	B-72 h	9.9, ±6.8	Very likely beneficial
Peak Torque 180°/s Dominant leg flexion	B-24 h	12.4, ±13.8	Likely beneficial
	B-48 h	8.0, ±18.9	Unclear
	B-72 h	10.2, ±12.1	Likely beneficial
RFD (0–200 ms)	B-24 h	22.7, ±31.7	Likely beneficial
	B-48 h	21.6, ±43.4	Unclear
	B-72 h	24.0, ±46.0	Unclear
CMJ	B-24 h	9.9, ±6.2	Very likely beneficial
	B-48 h	8.7, ±6.9	Likely beneficial
	B-72 h	6.1, ±4.7	Likely beneficial
RSI	B-24 h	2.6, ±11.3	Unclear
	B-48 h	2.6, ±15.1	Unclear
	B-72 h	5.6, ±12.2	Unclear
5 m sprint	B-24 h	−2.7, ±3.7	Possibly beneficial
	B-48 h	−3.3, ±5.3	Unclear
	B-72 h	−0.8, ±3.6	Unclear
10 m sprint	B-24 h	−1.4, ±3.5	Unclear
	B-48 h	−1.8, ±3.9	Unclear
	B-72 h	−0.5, ±3.9	Unclear
20 m sprint	B-24 h	−1.5, ±2.6	Unclear
	B-48 h	−1.3, ±2.9	Unclear
	B-72 h	0.3, ±2.2	Unclear

^a^ Mean effect refers to MILK minus CHO; ^b^ ± 90% CI: add and subtract this number to the mean effect to obtain the 90% confidence intervals for the true difference; ^c^ Qualitative Inference represents the likelihood that the true value will have the observed magnitude.

**Table 2:** Effects on soreness, tiredness, symptoms of stress, CK and hsCRP following repeated sprinting and jumping.

Variable	Time Frame	Mean Effect ^a^, ±/x/÷ 90% CI ^b^	Qualitative Inference ^c^
Muscle soreness (Squat)	B-24 h	−1.7, ±2.8	Unclear
	B-48 h	−1.1, ±2.1	Unclear
	B-72 h	−0.7, ±1.2	Unclear
Muscle soreness (Isokinetic knee extension/flexion)	B-24 h	−1.4, ±2.2	Unclear
	B-48 h	−1.1, ±2.3	Unclear
	B-72 h	−1.0, ±2.6	Likely beneficial
Muscle tiredness	B-24 h	−2.0, ±3.0	Unclear
	B-48 h	−1.6, ±3.2	Unclear
	B-72 h	−1.4, ±2.3	Unclear
DALDA B	B-24 h	−2.0, ±2.4	Very likely beneficial
	B-48 h	−1.8, ±3.0	Likely beneficial
	B-72 h	−1.0, ±2.6	Unclear
CK	B-2 h	1.1, x/÷1.3	Unclear
	B-24 h	1.1, x/÷1.6	Unclear
	B-48 h	1.3, x/÷ 1.8	Unclear
	B-72 h	1.3, x/÷1.8	Unclear
hsCRP	B-2 h	1.1, x/÷1.2	Unclear
	B-24 h	1.2, x/÷1.8	Unclear
	B-48 h	1.4, x/÷1.8	Unclear
	B-72 h	1.3, x/÷1.7	Unclear

^a^ Mean effect refers to MILK minus CHO; ^b^ ± 90% CI: add and subtract this number to the mean effect to obtain the 90% confidence intervals for the true difference; **^c^** Qualitative Inference represents the likelihood that the true value will have the observed magnitude.

## Supplementary Materials

The following is available online at http://www.mdpi.com/2072-6643/10/2/228/s1, Table S1: Within-group effects over time for dependent variables.
