# Supplementary material for: Milk: An Effective Recovery Drink for Female Athletes
Source: Nutrients. 2018 Feb 17;10(2):228. doi: 10.3390/nu10020228 (PMC5852804; doi:10.3390/nu10020228)
Supplement: Supplementary file 1 [file nutrients-10-00228-s001.pdf]

SUPPLEMENTARY MATERIAL

Table S1: Within-group effects over time for dependent variables

Variable	Timeframe	Mean effect, ±/ x/÷ 90% CI[a]	Qualitative inference[b]
**Peak Torque 60°/s Extension**			
MILK	B-24	-8.2, ±2.7	Most likely lower
	B-48	-8.5, ±2.8	Most likely lower
	B-72	-4.1, ±4.8	Most likely lower
CHO	B-24	-13.4, ±6.0	Most likely lower
	B-48	-12.5, ±7.0	Very likely lower
	B-72	-10.6, ±7.8	Very likely lower
**Peak Torque 60°/s Flexion**			
MILK	B-24	-5.2, ±8.8	Possibly lower
	B-48	-10.4, ±9.1	Likely lower
	B-72	-3.3, ±7.6	Unclear
CHO	B-24	-14.2, ±7.1	Very likely lower
	B-48	-13.3, ±5.4	Very likely lower
	B-72	-12.2, ±7.8	Very likely lower
**Peak Torque 180°/s Extension**			
MILK	B-24	-7.6, ±5.2	Likely lower
	B-48	-5.8, ±7.6	Likely lower
	B-72	-0.5, ±6.6	Unclear
CHO	B-24	-12.7, ±2.7	Most likely lower
	B-48	-14.5, ±7.4	Very likely lower
	B-72	-8.8, ±2.3	Most likely lower
**Peak Torque 180°/s Flexion**			
MILK	B-24	-2.8, ±8.0	Possibly lower
	B-48	-4.3, ±9.1	Possibly lower
	B-72	-1.6, ±9.3	Unclear
CHO	B-24	-14.0, ±7.7	Very likely lower
	B-48	-11.7, ±13.2	Likely lower
	B-72	-10.8, ±6.1	Very likely lower
**CMJ**			
MILK	B-24	-1.2, ±3.4	Unclear
	B-48	-1.9, ±3.1	Possibly lower
	B-72	-0.6, ±1.4	Very likely trivial
CHO	B-24	-10.6, ±4.2	Most likely lower
	B-48	-9.9, ±5.0	Very likely lower
	B-72	-6.6, ±4.0	Very likely lower

**RSI**			
MILK	B-24	-7.0, ±5.2	Likely lower
	B-48	-12.8, ±7.0	Very likely lower
	B-72	-5.3, ±8.0	Possibly lower
CHO	B-24	-10.8, ±9.0	Likely lower
	B-48	-14.2, ±10.8	Likely lower
	B-72	-10.8, ±7.8	Likely lower
**5m sprint**			
MILK	B-24	-1.7, ±2.4	Possibly lower*
	B-48	0.4, ±4.2	Unclear
	B-72	-0.6, ±3.3	Unclear
CHO	B-24	-4.8, ±3.2	Very likely lower*
	B-48	-3.1, ±2.9	Likely lower*
	B-72	-1.4, ±2.0	Possibly lower*
**10m sprint**			
MILK	B-24	-1.2, ±2.1	Possibly lower*
	B-48	-0.1, ±2.7	Unclear
	B-72	-0.3, ±3.2	Unclear
CHO	B-24	-2.4, ±3.0	Likely lower*
	B-48	-1.9, ±2.8	Unclear
	B-72	-0.5, ±2.8	Unclear
**20m sprint**			
MILK	B-24	-1.3, ±1.4	Possibly lower*
	B-48	-0.6, ±1.3	Possibly lower*
	B-72	-0.6, ±1.4	Possibly lower*
CHO	B-24	-2.7, ±2.3	Likely lower*
	B-48	-2.2, ±2.8	Unclear
	B-72	-0.1, ±1.7	Unclear
**RFD**			
MILK	B-24	-11.1, ±11.9	Possibly lower
	B-48	-20.0, ±9.9	Very likely lower
	B-72	-9.9, ±14.5	Possibly lower
CHO	B-24	-23.0, ±9.3	Most likely lower
	B-48	-24.7, ±12.7	Very likely lower
	B-72	-18.5, ±16.4	Likely lower
**Creatine Kinase**			
MILK	B-24	2.2, x/÷ 1.4	Most likely increased
	B-48	1.8, x/÷ 1.5	Very likely increased
	B-72	1.6, x/÷ 1.5	Likely increased
CHO	B-24	2.1, x/÷ 1.3	Most likely increased
	B-48	1.4, x/÷ 1.5	Likely increased
	B-72	1.1, x/÷ 1.4	Unclear

**hsCRP**			
MILK	B-24	1.2, x/÷ 1.8	Unclear
	B-48	1.2, x/÷ 1.7	Unclear
	B-72	1.1, x/÷ 1.8	Unclear
CHO	B-24	1.0, x/÷ 1.2	Unclear
	B-48	0.9, x/÷ 1.2	Likely lower
	B-72	0.8, x/÷ 1.3	Possibly lower
**Soreness**			
MILK	B-24	5.3, ±1.6	Most likely increase
	B-48	4.3, ±1.5	Most likely increase
	B-72	2.0, ±0.7	Most likely increase
CHO	B-24	5.8, ±1.3	Most likely increase
	B-48	4.8, ±1.1	Most likely increase
	B-72	2.6, ±0.8	Most likely increase
**Tiredness**			
MILK	B-24	4.3, ±1.1	Most likely increase
	B-48	3.8, ±1.0	Most likely increase
	B-72	1.8, ±0.8	Very likely increase
CHO	B-24	5.1, ±0.9	Most likely increase
	B-48	3.8, ±1.2	Most likely increase
	B-72	2.3, ±0.8	Most likely increase
**DALDA**			
MILK	B-24	3.4, ± 1.2	Most likely increase
	B-48	3.9, ±1.7	Most likely increase
	B-72	2.8, ±1.9	Very likely increase
CHO	B-24	4.7, ±1.7	Most likely increase
	B-48	4.9, ±2.2	Most likely increase
	B-72	3.2, ±1.6	Very likely increase

*[a]± 90% CI: add and subtract this number to the mean effect to obtain the 90% confidence intervals for the true difference; [b]Qualitative Inference represents the likelihood that the true value will have the observed magnitude; *lower refers to reduced performance*
